# Uptake of genetic counseling, genetic testing and surveillance in hereditary malignant melanoma (*CDKN2A*) in Norway

**DOI:** 10.1007/s10689-016-9939-8

**Published:** 2016-11-01

**Authors:** Trine Levin, Lovise Mæhle

**Affiliations:** Section on Hereditary Cancer, Oslo University Hospital, PB 4950 Nydalen, 0424 Oslo, Norway

**Keywords:** Hereditary malignant melanoma, *CDKN2A*, Uptake, Genetic counseling, Genetic testing, Surveillance

## Abstract

Germline mutations in the *CDKN2A* gene are associated with an increased risk of malignant melanoma and pancreatic cancer. In order to find out if the behavior pattern in families with a *CDKN2A* mutation is similar to what we previously have described in families with a *BRCA1* mutation, we have studied the uptake of genetic services in probands and their relatives. We describe whether they attend genetic counseling when invited, whether they want a mutation test after being counseled and whether they adhere to recommendations for surveillance. 66 % (95/144) of first-degree relatives to mutation carriers contacted us within the study period. 98 % (126/128) of all relatives who came for genetic counseling decided on genetic testing for their family’s mutation, and 93 % (66/71) of all mutation carriers wanted referral to yearly skin examinations. Female relatives had a significantly higher uptake of genetic services compared to males, similar to the findings in families with a *BRCA1* mutation. Uptake of genetic services in general in families with a *CDKN2A* mutation is high. Females seem to have a higher interest in genetic testing than males, regardless of gene mutated.

## Introduction

For many cancer syndromes there are established surveillance guidelines. They have been extensively evaluated and many have been proven efficient. The goal is prevention, early diagnosis and cure. The cancer syndromes caused by germline mutations in *CDKN2A* and *CDK4* have not been studied to the same degree. In our cancer genetic clinic we provide genetic counseling, genetic testing and follow-up for all hereditary cancer syndromes. We wish to determine whether we reach all at-risk individuals in families with a *CDKN2A* mutation in order to inform them of the possibility of genetic testing and surveillance.

After the identification of *CDKN2A* [1] and *CDK4* [2] genetic testing of families with hereditary malignant melanoma is possible. Genetic testing for disease-causing mutations has been available for many years for numerous cancer syndromes and uptake of genetic testing has been evaluated for several of these. In breast and ovarian cancer families, for example, early evaluation of uptake of genetic testing ranged from 43 to 80 % [3–5]. In our own clinic, studying genetic testing for a known *BRCA1* mutation in a population of at-risk females, 30 years or older, showed that 82 % pursued genetic testing [6]. In some reports, including ours, females were shown to be significantly more interested in genetic testing than males [4, 6–8]. This was believed to be due to the higher cancer risks for women in these families.

Having mutations in of *CDKN2A* and *CDK4* is associated with a significant increased risk of malignant melanoma. Early diagnosis is crucial for the prognosis of cutaneous malignant melanoma (CMM). The actual life-time risk of developing CMM among mutation carriers is high, but varies greatly due to many factors including other host characteristics and geographical differences [9]. This is related to both genetic and environmental factors. For example, the pigmentation gene *MC1R* acts as a modifier gene and influences the risk of malignant melanoma in both carriers and non-carriers [10]. Norway has among the highest incidences in the world of malignant melanoma with an accumulated risk up to the age of 75 of 2.4 % for both genders [11], while mutations in *CDKN2A* and *CDK4* were only found in 6.9 % in a population-based study among individuals with multiple primary melanomas (MPM) [12]. As a result, there are many families in our clinic that show numerous cases of malignant melanoma probably due to shared skin types and shared tanning practices where we cannot identify a mutation.

There are different observations of which other cancer types are associated with mutations in *CDKN2A*. Goldstein et al. [13] found that the risk of pancreatic cancer for *CDKN2A* mutation-carriers was 22-fold compared to the general population. This increase was not observed in melanoma kindreds without a demonstrated mutation. The Dutch founder mutation in *CDKN2A*, called “p16-*Leiden*”, is reported to confer the highest known lifetime risk of developing pancreatic cancer of about 17 % [14]. On the other hand, pancreatic cancer was not found to be associated with *CDKN2A* mutations in a GenoMel study from Australia [15]. This could be caused by genotype/phenotype differences. There are several reports of increased risks of other cancer types in *CDKN2A*-families in addition to pancreatic cancer [16–19]. One prospective study from Sweden found an especially increased risk of cancer of the pancreas, upper digestive and respiratory tissues in carriers of the Swedish founder mutation (p.Arg112dup) who had smoked [18]. Potjer et al. [19], through another prospective study of the p16-*Leiden* cohort, found an increased risk among tobacco users of cancers in the pancreas, respiratory tract and head and neck.

We have previously reported on information-flow in families with *BRCA1* mutations [20]. At that time, there had recently been passed a law in Norway determining that health workers were not allowed to approach and contact potential carriers of genetic mutations. The reported study was undertaken for the purpose of evaluating this law. We examined whether genetic information reached those at risk by only inviting other family members to contact us through our patients. We found that in our population, only 3 % of at-risk adult females did *not* contact us during the study period (conferring a 97 % uptake of genetic services). After communicating with our patients, it was established that the 3 % had, indeed, been given the information of our services. This indicated that it was the non-responders’ own decision not to have contacted us and that the system of having the patients inform their own relatives functioned as a way of spreading information throughout families.

Adherence to surveillance recommendations has been studied in many hereditary cancer syndromes. In a report by Stoffel et al. [21] of Americans in families with colorectal cancer with or without an identified mismatch-repair (MMR) mutation, 73 % followed their recommended program of colonoscopies every two years. The data was gathered through self-reported questionnaires. There is a possibility that responders to the questionnaire have a higher compliance rate than those who chose not to reply. Li-Fraumeni syndrome (LFS) is different in that there are no established complete periodic screening recommendations due to the range of tumor locations and ages of onset. Yet, in a study on subjects with at least a 50 % risk of having LFS, 78 % of them responded that they complied with the screening options that had been recommended to them. The type of screening was not universal, but organ-targeted screening based upon the cancers in their family. This study was also based upon self-reporting [22].

There are few publications on surveillance in families at risk of CMM. In a report from Australia [23], 166 *CDKN2A* mutation carriers were asked about their surveillance practices, both skin self-examination and clinical skin-examination. Overall, 21 % of them performed skin self-examination monthly, while 43 % reported having had a clinical skin-examination in the last 12 months. Only 17 % adhered to their recommendations of performing clinical skin-examination twice a year.

In a study published in 2008, Aspinwall et al. [24] showed that the intention to undergo both clinical screening and self-examination increased along with photo-protection behavior after disclosure of *CDKN2A* mutation status. In that study, the participants had previously been informed of the benefits and recommendations of regular screening. Still, before disclosure of their genetic results, only 52 % had received a total body skin examination within the last year. Those with at personal history of melanoma were more compliant at 78 %. Learning of their mutation status improved the overall intention to follow recommendations of total body skin examination and skin self-examination in all groups. Even among the mutation-negative participants, compliance with the recommended skin self-examination increased; thus, undermining the notion that a normal test result will give a false sense of security.

In the current study, we compare our knowledge of families with a *CDKN2A* mutation with our previous experiences in families with *BRCA* mutations. We describe uptake of contacting us as a way to gauge whether our methods of communication with our patients in the clinic also work in families with hereditary malignant melanoma (uptake of genetic counseling). It is also a goal of the current report to study individuals’ interest in genetic testing (uptake of genetic testing). We look at the differences in these parameters between males and females, since mutations in *CDKN2A*, as far as we know, give similar CMM risks to both genders, unlike *BRCA* mutations. We also wish to determine if carriers of *CDKN2A* mutation follow our recommendation of yearly dermatological examinations (uptake of surveillance).

## Materials and methods

### Patients and recruitment

The probands included in this study had all been diagnosed with at least two primary malignant melanomas and they all carried a mutation in *CDKN2A*. They were referred to us in February 2008 after the conclusion of a separate, population-based project which aimed to describe the prevalence of hereditary malignant melanoma in Norway. The inclusion criteria and results of that project were described in detail elsewhere [12]. In short, all living individuals who had been registered in the Cancer Registry of Norway with MPM between the years 1953 and 2004 were invited to participate. Blood was collected for genetic testing of *CDK4* and *CDKN2A*. Disease-causing mutations were found in 6.9 % [12]. Following the conclusion of that project, nineteen *CDKN2A* carriers were referred to us for genetic counseling and follow-up. This cohort will be referred to as probands. Once they were referred to us, they were incorporated into our regular cancer genetics clinic. All persons in our clinic are referred to as patients, regardless of disease status. Two among the 19 probands were father and son, so in total there were 18 separate families with 7 different disease-causing mutations in *CDKN2A*. Those with mutations in *CDK4* were referred to another genetics clinic in Western Norway and were not included in the current study.

### Probands

After the 19 probands had been referred to us, we sent them a letter with an appointment for genetic counseling. All had previously been informed by the dermatologist running the project that they harbored a mutation in *CDKN2A* [12]. Genetic counseling sessions included drawing a detailed family history, discussing basic relevant genetics and inheritance, sharing information on risks associated with carrying a mutation in *CDKN2A*, including that of pancreatic cancer risk and sun-tanning behavior. Probands also received the option of annual dermatological examinations. In addition, we received written permission to confirm relevant diagnoses from hospital files or the Cancer Registry of Norway, both for the probands and their deceased, affected relatives. During the first counseling session, we identified the relatives that should be informed of the possibility of genetic counseling, genetic testing and potentially dermatological surveillance. The probands were given sheets with information to give to these relatives and contact forms for them to fill out and return to us. We are, by law, not allowed to contact at-risk persons who have not been in touch with us first and we are, therefore, dependent upon the patient to reach and inform their own family members. We did not make assumptions regarding from which side of the family the mutation may have originated, but we started on the side affected with malignant melanoma or pancreatic cancer. At that first consultation, a blood sample was collected to confirm the proband’s carrier status.

Five out of the 19 probands either did not show up for genetic counseling or cancelled their appointment. A phone call to each of them revealed different reasons for not wanting an appointment. During the phone interview we drew a family history and identified the relatives who should receive information regarding our services. All probands indicated that they would contact these relatives. Following the conversation, information and documentation were mailed to these five probands. They also received information and contact materials to pass on to their relatives.

### Relatives

The relatives who contacted us by returning the form given to them by the probands were given genetic counseling. Their place in the pedigree was determined and the risk of carrying the particular mutation in *CDKN2A* established. We counseled them of relevant genetics, the risks associated with the mutation and sun-exposure. The relatives were also informed of the potential risk of pancreatic cancer and of our inability to provide efficient regular surveillance of the pancreas to ensure early diagnosis.

All patients, both probands and their relatives, were counseled by the same genetic counselor. All were given the same information regarding cancer risk for both malignant melanoma and pancreatic cancer. Only one family carried a truncating mutation, yet the information given was not differentiated.

All were offered genetic testing, except those coming to the counseling session together with a parent who had not yet been tested. When the genetic tests were completed, all received a neutral letter for a new appointment. They had to return to the clinic for their results, where another blood sample was tested for confirmation of the genetic results. These elements are standard procedures in our clinic.

### Uptake definition

**Fig. 1:**
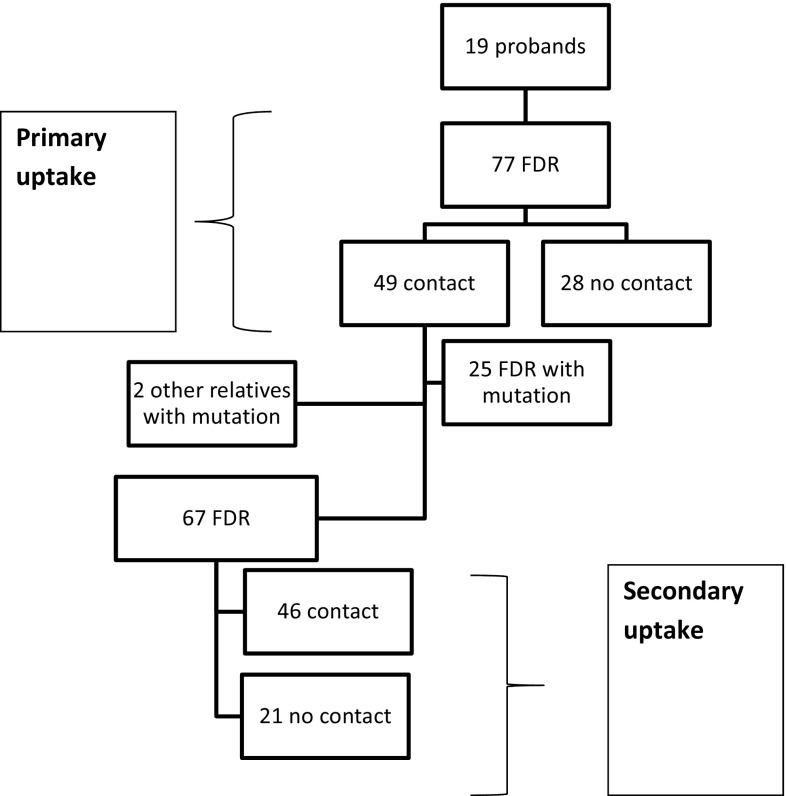
Uptake of genetic counseling

We determined the primary and secondary uptake of genetic services. We defined “primary uptake” as the proportion of the probands’ first-degree relatives (FDR) (alive and above 18 years of age) who contacted us. When we tested all the relatives who contacted us, some turned out to harbor the mutation in the family. Once they knew of their results, they became the new index person in a “secondary uptake”. They, in turn, informed their own FDR of the possibility of genetic testing, counseling and surveillance. The fraction of those new FDR who contacted us was defined by us as the “secondary uptake”. Each person was only counted once, so, for example, a proband was not counted as a relative to the new index person, even if they may be FDR. A flowchart depicting primary and secondary uptake is shown in Fig. 1.

We only included those index persons who had been given their genetic testing results prior to December 31, 2010, and counted only the relatives who contacted us prior to December 31, 2011. Thereby, allowing a minimum of one year for a relative of an index person to contact us.

We were very strict with the inclusion criteria for uptake. There were cases where a potential FDR did not contact us, but his/her children contacted us instead. The reason could have been that the FDR was ill, old or deceased; we still did not include the children as FDR, and they were not counted in our uptake of genetic counseling estimates. Only if the children turned out to be mutation carriers were they counted and then only as index persons in our “secondary uptake”.

### Surveillance

In contrast with many of the reports on surveillance, we refer our own patients to their initial follow-up appointment, thereby, not relying on the patients or other physicians to do so.

Probands and their relatives with mutations were offered and referred to yearly skin examinations at a regional hospital dermatological department. For many, this required traveling long distances. In many cases, we were able to find a satisfactory surveillance program closer to their home. We plan to report on the results of the surveillance of our patients at a later date.

### Statistical analysis

To explore the level of significance of the observed differences between the groups (Table 2), we used *χ* ^2^ test (IBM SPSS Version 22).

### Ethics

All activities were part of the Norwegian public health-care system. All who proceeded with a genetic test were required to sign an informed consent form, as is standard for all predictive testing in a clinical setting in Norway. All patient materials were kept in our medical files. No named information was exported from the medical files and no separate research registry including patient names was established.

## Results

By December 31, 2011, the 19 probands from 18 kindreds had been counseled and instructed to give information to their relatives about the possibility of genetic services. 133 of them had stepwise contacted us in writing. This included FDR to our probands, but there were also others who were more distantly related. All were given an appointment for genetic counseling.

An overview of all the mutation carriers, their mutations and information regarding the melanomas and pancreatic cancers is depicted in Table 1.

**Table 1:** Distribution of melanomas and pancreatic cancer among *CDKN2A* mutation carriers

Mutation		# Families	# Mut. carriers (probands)	# Carriers with melanoma (probands)	# Melanomas (probands)	MM-age of onset-range (probands)	Pancreas (#)
c.79G > T	p.Glu27X	1	7 (1)	2 (1)	4 (2)	47–55 (47–51)	
c.159G > C	p.Met53Ile	7	33 (7)	11 (7)	32 (22)	31–72 (31–68)	Panc (2)
c.242C > G	p.Pro81Arg	2	9 (2)	6 (2)	9 (6)	33–64 (41–64)	Panc (2)
c.259C > T	p.Arg87Trp	2	2 (2)	2 (2)	5 (5)	28–53 (28–53)	
c.353C > T	p.Ala118Val	4	15 (4)	5 (4)	12 (11)	34–79 (40–79)	
c.379G > C	c.Ala127Pro	1	2 (2)	2 (2)	7 (7)	34–76 (34–76)	
c.392G > C	c.Arg131Pro	1	7 (1)	4 (1)	6 (3)	16–38 (19–38)	

### Uptake of genetic counseling

The probands had 77 FDR of which 49 contacted us (64 %) during the study period (Fig. 1). Our secondary uptake showed that 46 out of a possible 67 FDR of the emerging new mutation carriers contacted our clinic (69 %) (Fig. 1). There was no significant difference between the primary and secondary uptake (*p* > 0.5) and we, therefore, combined the two groups (Table 2; Fig. 1). Combining the primary and secondary uptakes, we obtained a total uptake of 66 % (95/144).

**Table 2:** Uptake of genetic counseling with gender distribution

	Contact (%)			No contact (%)			Total
	1° Uptake	2° Uptake	Total	1° Uptake	2° Uptake	Total	
Females	29 (71)	31 (84)	60 (77)	12 (29)	6 (16)	18 (23)	78
Males	20 (56)	15 (50)	35 (53)	16 (44)	15 (50)	31 (47)	66
Total	49 (64)	46 (69)	95 (66)	28 (36)	21 (31)	49 (34)	144

Seventy-seven percent of the female FDR compared to 53 % of the males had contacted us by the end of the study period, thereby showing a highly significant gender difference (*p* = 0.003; Table 2).

### Uptake of genetic testing and genetic results

All of the 133 relatives who contacted us seeking genetic services were given a genetic counseling appointment. Of these, 128 were offered genetic testing. Those who were not offered testing were individuals where either their parents turned out not to carry the family’s mutation or they did not turn up for their genetic counseling appointment. Only two individuals at risk decided that they did not want genetic testing following genetic counseling, giving a 98 % uptake of genetic testing (126/128).

**Fig. 2:**
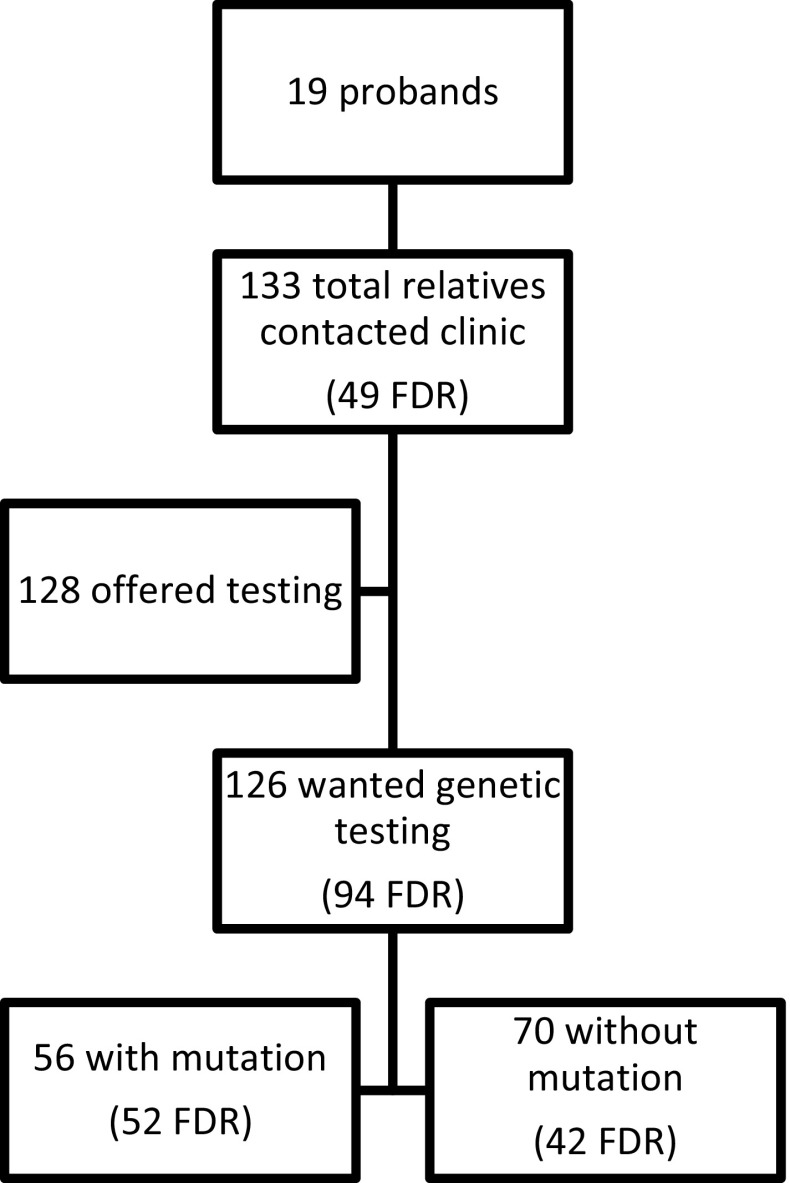
Uptake of genetic testing

In total, 70 received results that they did not carry the mutation in their family, while 56 harbored the mutation in *CDKN2A*. Among all FDR, 95 individuals, only one decided not to proceed with genetic testing. Of the remaining 94, 52 (55 %) individuals had the mutation, while 42 (45 %) found out that they did not carry the mutation found in their FDR. Uptake of genetic testing and the results of testing are shown in Fig. 2.

### Uptake of surveillance (compliance)

All nineteen probands had the opportunity to attend our regular surveillance program. Three had died, two of which from pancreatic cancer. Three probands did not, to our knowledge, attend any surveillance program. Two of them had cancelled their appointment, while one indicated he was too old. One was followed by her primary doctor due to an unrelated, debilitating condition. Three decided to continue surveillance at a local hospital. In total, thirteen probands wanted follow-up, which indicated a total compliance of 81 % (13/16).

By December 31, 2011, 56 relatives had been tested and determined to be mutation carriers of the family’s mutation in *CDKN2A*. In this group, two declined the option of yearly skin surveillance. In the remaining group, one had died of malignant melanoma diagnosed prior to our contact. Another person lived abroad and received a letter in English from us explaining our surveillance recommendations. One had attended appointments on his own as part of the follow-up of his malignant melanoma diagnosis, also previously diagnosed. Referrals were given to the remaining individuals. With only two mutation-positive relatives declining follow-up, compliance with surveillance in this group was 96 % (53/55). The results of the uptake of surveillance are shown in Table 3.

**Table 3:** Uptake of surveillance

	Surveillance (%)	No surveillance	Total
Proband	13 (81)	3	16
Relatives (Mut+)	53 (96)	2	55
Total	66 (93)	5	71

All FDR with a personal history of malignant melanoma contacted us within the time of the study and all proceeded with genetic testing. All had the mutation in the family.

## Discussion

In this study of families with a *CDKN2A* mutation, we have reported their interest in genetic services by studying if they come when invited, if they want genetic testing and whether they adhere to our surveillance program. In general, we found a relatively high uptake across all parameters compared to studies published from other populations.

### Uptake of genetic counseling

During our study period, 66 % (95/144) of all FDR of mutation carriers contacted us (overall uptake of genetic counseling). Originally, we had expected that the first group of FDR (primary uptake), those closest to the probands, would be more motivated to contact us than the next wave of relatives. All in the first group would, by definition, have a close relative with at least two bouts of malignant melanoma, which was not always the case in the second group. We had, therefore, separated our FDR into two groups, those who were FDR of our initial probands (primary uptake) and those who were FDR to the emerging group of mutation carriers (secondary uptake). These two groups turned out not to be significantly different with regard to uptake and gender distribution, and they were, therefore, combined (Table 2). One reason why these two groups are not as different as anticipated, might be that the presence of disease is not the determining factor why people seek genetic services, but the knowledge of the presence of the mutation is, which is equal in both groups.

The uptake of genetic counseling is high but still much lower than our previous experience in *BRCA1* families (97 %) [6]. The reasons for this discrepancy could be many, but may have something to with the way the initial participants were ascertained. The current investigation originated from a research project [12]. The participants were contacted by researchers and asked if they wanted genetic testing based on the fact that they had developed MPM. The *BRCA* study, on the other hand, originated from individuals who had initiated contact with our clinic themselves searching for help to determine the cause of breast and ovarian cancer in their families.

Another reason why the *CDKN2A* and *BRCA* families behaved differently may be that all the probands in the current study had, by definition, survived their cancers. In fact, most affecteds in these families had not died from their diagnoses of malignant melanoma, It is possible that there was a perception that malignant melanoma is not such a “dangerous” condition, thus genetic testing was not that important. This is in contrast with our *BRCA1* families where a large percentage of the affected relatives had died from their disease.

There were mutation carriers where none of their FDR had contacted us. We knew from our phone interviews with those probands the number and gender of their FDR. Since these were large families, we found it strange that no-one had been interested in our services. We suspected that our contacts had not informed their relatives, thereby not allowing these relatives to decide for themselves whether to contact us or not. There was also one index person in our secondary uptake, where no-one from his large family contacted us. Because of the large sizes of these families, this reduced our combined uptake of genetic counseling drastically. If we had omitted the FDR from these three probands and one index person, the total uptake would have increased from 66 to 80 % (95/119).

We have, indeed, after the project was concluded, been informed that the relatives of one of the three probands whose families had not contacted us, found the papers from our clinic after the proband’s death. These relatives confirmed that their uncle had never mentioned anything to them about the presence of a mutation in their family.

The probands whose families did not contact us were all part of the group who chose not to come to the genetic counseling session in person. They only received a phone call. They had indicated that they would inform their relatives of our services, but did not. It may be that those patients who cancelled their opportunity to talk to us in person were, in fact, never interested, neither for their own sake nor for their relatives; thereby not letting their relatives decide for themselves. We did not contact our patients to question them as to the reasons we had not heard from any of their relatives. All of those individuals whose relatives did not contact us had been diagnosed with malignant melanoma.

How and if genetic information is communicated within a family is related to many aspects. It will be influenced by the proband’s own perceptions of the disease and how well it may be diagnosed and treated. It will also be influenced by the relationship to his/her family members, where close relations, both biological and/or social, are more likely to be informed and in the end, tested for the mutation in the family.

Our experiences in the current study in *CDKN2A* families have both similarities and differences from our own previous observations and conclusions in *BRCA1* families [6, 20]. In both groups the uptake was higher than described in most other reports. However, we cannot assume that the information about our services and the potential of risk reached all FDR even though our contacts indicated that they would inform their next of kin. These may be examples of “passive non-disclosure”, first described by Gaff et al. [25, 26] in families with HNPCC (hereditary non-polyposis colon cancer). These are situations in genetic counseling where patients imply that they will communicate information regarding genetic risk to their relatives, but do not. This is different from “active non-disclosure” where patients openly admit that they do not intend to contact other family members. Gaff et al. [25, 26] indicated that this may have different causes including that the index person assumes that the information is not relevant to some or all family members.

We found a significant gender difference in uptake of genetic counseling and females were more likely to contact us. We saw the same trend in families with a *BRCA1* mutation, but had concluded at that point that the probable reason was the cancer-risk difference. Male *BRCA1*-carriers have a close to normal cancer risk. Female carriers, on the other hand, have a significant risk of developing breast or ovarian cancer during their lifetime. We offer them information and referrals to different procedures, including prophylactic surgeries, which will significantly increase their chance of survival. With *CDKN2A*, on the other hand, there is no known gender difference with regard to cancer risk. The gender difference in uptake may, therefore, show that females are, indeed, more likely to seek health services when indicated, or at least, that they do so more promptly. The reasons for this may be many, but one might be that women often feel more responsible for the health of her family members and herself. Females have previously been named the “housekeepers” of genetic information in the family, in that they talk more about cancer in the family and are the main keepers of information even when the genetic risk is on their husbands’ side [27].

### Uptake of genetic testing

Even though 66 % uptake of genetic counseling among FDR is low compared with 97 % among at-risk females in *BRCA* families, almost all who did contact us for the current study proceeded with genetic testing. This may indicate that in this current group, most made their decision whether to proceed with genetic testing or not, prior to contacting us. They may not perceive genetic counseling as a deciding factor in the decision-making process. It seems that the information given to them through the genetic counseling process confirmed their decision to pursue testing. This is different from our families with a *BRCA1* mutation where 97 % contacted us, while only 82 % of them pursued testing. This difference could also be a measure of how genetic testing has become more accepted in more recent years.

In a publication from Australia, Forrest et al. [28] looked at the uptake of genetic testing in four non-cancer syndromes with different heritance patterns; chromosomal translocations, fragile X syndrome, Huntington syndrome (HD) and spinal muscular atrophy. They found that uptake of genetic testing among FDR spanned from 45 % for HD to 79 % for Fragile X. This shows that the genetic condition and its implications do make a difference when it comes to decisions on whether to proceed with genetic testing. Interestingly, Forrest et al. saw only a gender difference in uptake of testing for Fragile X, where females were significantly more likely to pursue testing to learn of their genetic status; not surprising given the X-linked inheritance pattern with this condition [28].

### Uptake of surveillance

We see that uptake of surveillance with annual skin examinations by a dermatologist at a university hospital was very high as compared with other studies [23, 24]. In this study, the highest adherence was observed among the mutation-positive relatives (96 %), compared to the probands (81 %). It may be surprising that not all of the probands, who by definition have had malignant melanoma at least twice, wished to be examined regularly, but many in the group that declined follow-up referred to their old age as a contributing factor. The high adherence to uptake of surveillance might be related to the fact that health treatment and follow-up are paid for by the state in Norway and that our patients trust that the program for following moles is effective in preventing malignant melanoma. Skin examinations are also non-invasive and pain-free in comparison to the screening for many of the other hereditary cancers.

### Study limitations

In our group of relatives there were both individuals with a previous diagnosis of malignant melanoma (and other cancers) and individuals without cancer. Ascertainment may have been influenced by the possibility that affecteds were more likely to contact us when invited than others. All relatives with a personal history of malignant melanoma who contacted us had the mutation in the family.

We do not know whether our findings are representative for other populations since these findings are dependent upon how genetic services are funded and upon family structures.

## Conclusions

In conclusion, we have found there to be a generally high uptake of all aspects of genetic services in *CDKN2A*-families as they were in *BRCA1* families. Our results indicate that most people seek a resolve regarding genetic status even if we are not able to provide surveillance for all cancer risks, such as pancreas cancer. Females were significantly more likely to participate regardless of the family having a mutation in *CDKN2A* or *BRCA1*. Even though our uptake was high there were a few individuals who did not seem to inform their relatives at all. We believe that all clinical work should systematically be evaluated and monitored. This will provide valuable and necessary knowledge for the future.
